# Impact of a diabetes pay-for-performance program on nonincentivized mental disorders: a panel study based on claims database analysis

**DOI:** 10.1186/s12962-023-00450-y

**Published:** 2023-07-06

**Authors:** Ming-Chan Sung, Kuo-Piao Chung, Shou-Hsia Cheng

**Affiliations:** 1 Institute of Health Policy and Management, College of Public Health, National Taiwan University, Taipei, Taiwan; 2 Population Health Research Center, National Taiwan University, 17, Xu-Zhou Road, Taipei, 100 Taiwan

**Keywords:** Diabetes, Pay-for-performance, Spillover effect, Depression, Disease management

## Abstract

**Background:**

Diabetes is one of the most prevalent chronic diseases with subsequent complications. The positive effects of diabetes pay-for-performance (P4P) programs on treatment outcomes have been reported. The program provides financial incentives based on physiological care indicators, but common mental disorder complications such as depression are not covered.

**Methods:**

This study employed a natural experimental design to examine the spillover effects of diabetes P4P program on patients with nonincentivized depressive symptoms. The intervention group consisted of diabetes patients enrolled in the DM P4P program from 2010 to 2015. Unenrolled patients were selected by propensity score matching to form the comparison group. Difference-in-differences analyses were conducted to evaluate the effects of P4P programs. We employed generalized estimating equation (GEE) models, difference-in-differences analyses and difference-in-difference-in-differences analyses to evaluate the net effect of diabetes P4P programs. Changes in medical expenses (outpatient and total health care costs) over time were analysed for the treatment and comparison groups.

**Results:**

The results showed that enrolled patients had a higher incidence of depressive symptoms than unenrolled patients. The outpatient and total care expenses of diabetes patients with depressive symptoms were lower in the intervention group than in the comparison group. Diabetes patients with depressive symptoms enrolled in the DM P4P program had lower expenses for depression-related care than those not enrolled in the program.

**Conclusions:**

The DM P4P program benefits diabetes patients by screening for depressive symptoms and lowering accompanying health care expenses. These positive spillover effects may be an important aspect of physical and mental health in patients with chronic disease enrolled in disease management programs while contributing to the control of health care expenses for chronic diseases.

## Background

Diabetes, as a chronic disease, can lead to severe complications over time. The global economic burden of diabetes will increase from U.S. $1.3 trillion in 2015 to $2.5 trillion by 2030, occupying a share of the global GDP ranging from 1.8% to a maximum of 2.2% [1]. The complications of diabetes affect patients’ morbidity and mortality and further increase health care costs [2]. To respond to these issues, pay-for-performance (P4P) programs are now extensively used for patients with diabetes. These programs develop criteria according to clinical practice guidelines and health outcomes. Health care providers are encouraged to provide guideline-recommended medical services for diabetes with additional financial incentives. Many studies evaluating diabetes P4P programs have shown substantial improvements in clinical care processes, medical utilization, costs, and outcomes [3–7]. Furthermore, diabetes P4P programs can lower the risk or delay the occurrence of diabetes-related physical complications [4, 8].

People with diabetes are more likely to have common mental disorders such as depression, anxiety, and stress [9, 10]. The number of diabetes patients with depression across their lifetime is 2 times greater than that of the general population [11]. Due to increased medical specialization, health care for diabetes patients with comorbid depression is insufficient and may even be worsening [12]. The American Diabetes Association (ADA) recommended that “psychosocial care should be integrated with collaborative, patient-centred medical care and provided to all people with diabetes, with the goals of optimizing health outcomes and health-related quality of life” [13]. According to the ADA 2023 “Standards of Care in Diabetes”, screening for psychosocial issues at diagnosis and during routine follow-up care could have an impact on diabetes management and provide appropriate referrals to trained mental health professionals [14]. The early screening and treatment of depression or diabetes-related stress can improve the health care outcomes of diabetes patients [15]. It should be a priority to identify and treat the mental health comorbidities in diabetes patients [11].

The Taiwan National Health Insurance (NHI) system was implemented in 1995. The NHI is a compulsory form of social insurance based on a single-payer model. It provides citizens with a universal, comprehensive benefit package covering inpatient and outpatient visits and the most common prescription drugs. For the medical care of diabetes patients, the Taiwanese NHI launched a diabetes mellitus P4P program (DM P4P) in 2001. The incentive indicators include case management fees, higher physician fees for patient follow-up visits, and specific payment items such as nutritional education, physical examinations, and laboratory tests. Clinical outcome indicators are also established, such as the maintenance of HbA1c levels below 7%. Physicians who meet the requirement set by the NHI can voluntarily participate in the DM P4P program.

For people with diabetes, early detection and appropriate treatment for depressive symptoms can reduce the emotional and financial burden of the disease. However, the design of incentive indicators for diabetes P4P programs focuses on diabetes care. Mental health conditions have seldom been taken into account in diabetes management programs. In the context of P4P programs, some unintended consequences have been observed in areas that were not subject to monitoring in P4P programs, including a substantial spillover effect into other areas of care for which financial incentives were not directly provided [16]. For the physical and mental health of diabetes patients, the impact assessment of the effect of the DM P4P programs on depressive symptoms is important. Spillover effects of chronic disease management may represent a major externality on psychological distress. The aim of this study was to explore the spillover effect of the DM P4P program on depressive symptoms under the NHI scheme in Taiwan.

## Methods

### Data source and study population

This study used a natural experimental design and employed longitudinal datasets relating to diabetes patients’ NHI claims in Taiwan. The database we used includes a nationally representative sample of patients diagnosed with type 2 diabetes mellitus from 2007 to 2015. To precisely select patients with diabetes from the NHI claims database [17], individuals with at least three diabetes-related outpatient visit records during 2007 and 2008 were defined as the diabetes population. The intervention group (n = 8,077) comprised diabetes patients who were first enrolled in the DM P4P program in 2009 as the baseline index. Each enrolled patient must have at least one DM P4P program record per year during the 6 follow-up periods from 2010 to 2015. Other diabetes patients who had never been enrolled in the program and had at least one medical claim record per year during the study period (2007 to 2015) were classified as the comparison group (n = 238,499) [18, 19].

In Taiwan, patient enrolment in the DM P4P program is at the discretion of the physician [20]. A previous study found that patients with more severe conditions or more comorbidities are more likely to be excluded from enrolment [21]. An enrolment selection bias may exist between the intervention and comparison groups [22]. To minimize the influence of selection bias and increase the homogeneity of subjects in the two groups, propensity scores were used to address systematic differences between patients who were enrolled and excluded from the DM P4P program [23].

We used logistic regression to generate propensity scores for each patient with diabetes in 2009. The calculation was based on related covariates [19], including patient sex, age group, Diabetes Complications Severity Index (DCSI) score [24], Charlson Comorbidity Index (CCI) score [25], and the accreditation level and location of the hospital visited.

To increase comparability, we used propensity score matching (PSM) to select proper subjects for the comparison group [26, 27]. PSM was conducted at the patient level using a 1:4 matching algorithm based on the abovementioned patient characteristics at baseline. After the PSM process, 8,073 patients enrolled in the P4P program were included as the intervention group and 30,514 unenrolled patients were included as the comparison group.

The DCSI comprises seven categories of diabetes complications with codes from 0 to 2 by severity, and scores range from 0 to 13. We used individual DCSI scores to group the patients into high (≥ 3), medium (2), and low diabetes severity (1 or 0) groups in this study. Based on CCI score criteria, comorbid disease was defined as at least one disease-specific ICD-9-CM code that appeared with reference to outpatient or inpatient services. In our analysis, CCI scores were grouped into three categories: 0, 1, and ≥ 2. For hospital accreditation, the levels were categorized as medical centre, regional hospital, district hospital, and community clinic, in descending order [28]. The location of the hospitals may adjust for the socioeconomic differences among regions in Taiwan [29]. The accreditation level and location of the hospitals that the subjects visited most frequently were identified in the analysis.

The study period was from 2007 to 2015 and started two years before the baseline index with six years of follow-up.

### Measures of study variables

To evaluate the incidence of depressive symptoms among diabetes patients after enrolment in the DM P4P program, patients with depression-related medical claim histories 2 years before the index date were excluded from the study population.

The documented diagnosis of depression was not always based on the diagnostic criteria for depressive disorder [30]. In this study, patients were considered to have depressive symptoms if they had at least one outpatient visit with a depressive disorder diagnosis or had one instance of antidepressant use during each follow-up year.

Outpatient expenses incurred by the patients were calculated for every follow-up year. Total health care expenses were defined as the sum of the costs for each patient, including ambulatory care, hospitalization, medication, and laboratory test costs, that appeared in the NHI claims records. Several confounding factors referenced in previous research were controlled for in the regression models, including sex, age group, CCI score, DCSI score, DM P4P program enrolment status, and hospital location and accreditation level [19].

### Statistical analysis

This study used a quasiexperimental design to examine the effect of the DM P4P program. We used multiple logistic regression models to estimate the effect of the DM P4P program on the occurrence of depressive symptoms in each follow-up year. For the comparison of outcome variables between the intervention and comparison groups, we employed generalized estimating equation (GEE) models to evaluate the net effect of P4P programs. To control unobserved variables in longitudinal data, difference-in-differences (DID) methods were used to measure the causal effect of nonrandom program intervention [31]. Changes in outcomes across the treatment and comparison groups over time were analysed. The DID estimation represented the difference in average outcomes in the treatment group before and after program enrolment minus the difference in average outcomes in the comparison group before and after program enrolment. To estimate the net effect of the DM P4P program, the changes in health care expenses (outpatient and total health care costs) of the diabetes patients were analysed as outcome variables between the two study groups before and after enrolment in the DM P4P program.

Moreover, to investigate the program effect on depressive symptom complications, health care expenses were further compared between diabetes patients with and without depressive symptom occurrence. We extended the analysis by using a difference-in-difference-in-differences (triple-difference) approach [32]. The average expense estimate of change over time for the diabetes patients with depressive symptoms enrolled in the P4P program, netting out changes over time in patients without depressive symptoms enrolled in the P4P program (first difference) and changes over time in the diabetes patients with depressive symptoms relative to diabetes patients without depressive symptoms in the unenrolled group (second difference) were analysed respectively. We took the difference in these two difference-in-differences estimators to evaluate the expense impact of the program on diabetes patients with depressive symptoms. The changes in outpatient care and total health care expenses were analysed to evaluate the spillover effect of the DM P4P program on depressive symptoms.

To further evaluate health care expenses in diabetes patients with depressive symptoms, we employed generalized estimating equation (GEE) models to analyse the outpatient expenses and total care expenses of depression-related care. The analyses were performed using SAS version 9.4 (SAS Institute, Cary, NC).

## Results

### Characteristics of diabetes patients before and after propensity score matching

Table 1 shows the baseline characteristics of the diabetes patients in the intervention and comparison groups before and after PSM. The inferences of the program effects using PSM are valid only if subjects in both the intervention and comparison groups have similar distributions of the measured baseline covariates [26]. The standardized mean difference (SMD) is the most commonly used statistic to assess the covariate balance of distributions between groups [33]. A standardized difference cut-off point larger than 0.10 can be considered as an indication of imbalance [34].

**Table 1:** Patient characteristics in the pre- and postmatched samples in 2009

		Prematched (N = 246,576)					Postmatched (N = 38,587)				
Variables		Intervention group		Comparison group		*P* value (SMD^c^)	Intervention group		Comparison group		*P* value (SMD^c^)
		n	%	n	%		n	%	n	%	
**Total**		**8,077**		**238,499**			**8,073**		**30,514**		
**Sex**						0.052					0.9246
	Male	3,929	48.6	118,636	49.7	(0.022)	3,929	48.7	14,833	48.7	(0.001)
	Female	4,148	51.4	119,863	50.3		4,144	51.3	15,681	51.3	
**Age group**						< 0.0001					0.9949
	< 55	2,639	32.7	62,857	26.4	(0.305)	2,638	32.7	10,001	32.8	(0.003)
	56–65	3,044	37.7	76,374	32.0		3,042	37.7	11,501	37.7	
	66–75	1,874	23.2	64,696	27.1		1,873	23.2	7,068	23.2	
	76+	520	6.4	34,572	14.5		520	6.4	1,944	6.4	
**DCSI** ^**a**^						< 0.0001					0.8229
	Score 0	3,723	46.1	123,712	51.9	(0.130)	3,723	46.1	14,227	46.6	(0.012)
	Score 1	2,342	29.0	57,070	23.9		2,340	29.0	8,809	28.9	
	Score 2	1,184	14.7	33,310	14.0		1,184	14.7	4,438	14.5	
	Score 3+	828	10.3	24,407	10.2		826	10.2	3,040	10.0	
**CCI** ^**b**^						0.0002					0.2217
	Score 0	4,688	58.0	134,882	56.6	(0.047)	4,688	58.1	17,976	58.9	(0.022)
	Score 1	2,448	30.3	72,229	30.3		2,446	30.3	9,175	30.1	
	Score 2+	941	11.7	31,388	13.2		939	11.6	3,363	11.0	
**Accreditation level of hospital**						< 0.0001					0.9607
	Medical centre	1,640	20.3	57,809	24.2	(0.474)	1,639	20.3	6,183	20.3	(0.007)
	Regional hospital	3,228	40.0	51,823	21.7		3,225	40.0	12,104	39.7	
	District hospital	1,369	17.0	33,846	14.2		1,369	17.0	5,213	17.1	
	Community clinic	1,840	22.8	95,021	39.8		1,840	22.8	7,014	23.0	
**Location of hospital**						< 0.0001					0.9314
	Taipei and northern	2,835	35.1	115,169	48.3	(0.296)	2,833	35.1	10,824	35.5	(0.008)
	Central	1,475	18.3	33,806	14.2		1,474	18.3	5,566	18.2	
	Southern/Kaoping	3,638	45.0	82,769	34.7		3,637	45.1	13,640	44.7	
	Eastern	129	1.6	6,755	2.83		129	1.6	484	1.6	

^a^ DCSI, Diabetes Complications Severity Index

^b^ CCI, Charlson Comorbidity Index

^c^ SMD, absolute value of the standardized mean difference

Before matching, significant differences were detected between the intervention and comparison groups with respect to most of the characteristics assessed. The absolute SMD values for age group, hospital accreditation level and hospital location were all more than 0.2. After PSM, all SMDs of covariates were less than 0.1. The variable distribution became more balanced between the intervention group and comparison group.

Among prematched study subjects, patients aged less than 65 years accounted for 70.4% and 58.4% of the intervention and comparison groups, respectively. After the PSM process, the percentage of patients aged less than 65 years in the comparison group increased to 70.5%. Among prematched patients, DCSI scores of 0 and 1 accounted for 51.9% and 23.9% of the comparison group, respectively. The percentages were 46.6% and 28.9% after matching, which is similar to the distribution of the intervention group. The postmatched study cohort consisted of 38,587 patients, with 8,073 and 30,514 patients in the intervention and comparison groups, respectively. Female patients accounted for 51.3% of the study subjects and approximately 58% of the patients had no comorbidities (CCI score = 0). The two study groups became more comparable.

### Effects of the DM P4P program on depressive symptoms

Table 2 presents the results based on multiple logistic regression models examining the occurrence of depressive symptoms among diabetes patients from 2010 to 2015. Diabetes patients enrolled in the P4P program were more likely to have depressive symptoms than unenrolled patients. Except for the second year of follow-up, there were significant differences in each year of follow-up. Female patients tended to have higher probabilities of depressive symptoms over the study period. The probabilities of depressive symptoms among the age groups followed a general upward trend over the study period. Diabetes patients with higher DCSI and CCI scores had a significantly higher probability of depressive symptoms. Furthermore, the likelihood of depressive symptoms was much higher in patients with the highest DCSI (3+) and CCI scores (2+) in those with lower scores (DCSI/CCI scores of 1 or less).

**Table 2:** The effect of the DM P4P program on depression onset from multiple logistic regression models in 2010–2015

		Year 1		Year 2		Year 3		Year 4		Year 5		Year 6	
Variables		β	SE	β	SE	β	SE	β	SE	β	SE	β	SE
**P4P status (ref: non-P4P)** Attend P4P		0.1728**	0.0657	0.0886	0.0497	0.1202**	0.042	0.1216**	0.0378	0.0915**	0.0353	0.083*	0.0334
**Sex (ref: Male)** Female		0.3538***	0.057	0.3021***	0.042	0.2953***	0.0356	0.2908***	0.0319	0.2657***	0.0295	0.27***	0.0278
**Age group (ref:<55 years)**													
	56–65	0.1031	0.071	0.1163*	0.0524	0.1711**	0.0445	0.1931***	0.0397	0.2057***	0.0367	0.2171***	0.0345
	66–75	0.2275**	0.0772	0.2609***	0.0571	0.3232***	0.0486	0.3527***	0.0435	0.3655***	0.0403	0.3897***	0.038
	76+	0.3581**	0.1113	0.4583***	0.0815	0.5225***	0.0697	0.5091***	0.0637	0.5152***	0.0596	0.5067***	0.0569
**DCSI (ref: Score 0)** ^**a**^													
	Score 1	0.389***	0.0682	0.3031***	0.0506	0.287***	0.0428	0.266***	0.0382	0.2602***	0.0352	0.2543***	0.0331
	Score 2	0.3341***	0.0851	0.3817***	0.0615	0.3867***	0.052	0.3658***	0.0467	0.3484***	0.0434	0.317***	0.0412
	Score 3+	0.6623***	0.0899	0.6663***	0.0672	0.6494***	0.0577	0.6123***	0.0526	0.6***	0.0493	0.5835***	0.047
**CCI (ref: Score 0)** ^**b**^													
	Score 1	0.2519***	0.0644	0.1577**	0.0476	0.1949***	0.04	0.194***	0.0358	0.1859***	0.0332	0.1967***	0.0313
	Score 2+	0.694***	0.081	0.5696***	0.0616	0.5419***	0.0534	0.5236***	0.0487	0.5186***	0.0457	0.4944***	0.0438
**Accreditation level of hospital (ref: Medical centre)**													
	Regional hospital	0.125	0.08	0.0428	0.0578	-0.00345	0.0483	0.00883	0.0435	0.026	0.0402	0.0284	0.0379
	District hospital	0.1533	0.0973	0.0848	0.0707	-0.0184	0.06	-0.00209	0.0539	-0.00717	0.0501	-0.0224	0.0473
	Community clinic	0.2259*	0.0878	0.122	0.0641	0.0477	0.054	0.0705	0.0485	0.064	0.045	0.0732	0.0424
**Location of hospital (ref: Taipei and northern region)**													
	Central region	0.0844	0.0792	0.0778	0.0586	0.0302	0.0502	0.0626	0.045	0.0551	0.0418	0.0299	0.0397
	Southern/Kaoping region	0.0254	0.0658	0.00777	0.0486	-0.00512	0.041	0.00396	0.0369	0.00784	0.0342	0.0106	0.0322
	Eastern region	0.2678	0.1959	0.1474	0.1543	0.0842	0.1348	0.1342	0.1203	0.1357	0.1125	0.1683	0.1058

* *P* < 0.05; ** *P* < 0.01; *** *P* < 0.001. ^a^ DCSI, Diabetes Complications Severity Index. ^b^ CCI, Charlson Comorbidity Index

### Effects of the DM P4P program on health care expenses

Table 3 shows the results of the P4P program on health care expenses from the generalized estimation equation model with DID analyses for each of the follow-up years. We measure the net effect of the P4P program by the parameters of the interaction term in the DID analysis. Changes in health care expenses as outcome variables were analysed to assess the impact of the DM P4P program interventions.

**Table 3:** Estimations of the impact of the DM P4P program on health care expenses: Difference-in-differences method

	Outpatient Expenses				Total Expenses		
Follow-up	Estimate	SE	*P* Value		Estimate	SE	*P* Value
Year 1	0.1819	0.0094	< 0.0001		0.0963	0.0149	< 0.0001
Year 2	0.1162	0.0093	< 0.0001		0.0833	0.0136	< 0.0001
Year 3	0.0715	0.0094	< 0.0001		0.0584	0.0134	< 0.0001
Year 4	0.0369	0.0098	0.0002		0.0228	0.0127	0.0735
Year 5	0.0032	0.0102	0.7564		-0.0055	0.0129	0.6714
Year 6	-0.028	0.0105	0.0078		-0.0336	0.0131	0.0103

Patients in the intervention group had significantly higher outpatient health care expenses in the first year. However, the net difference of estimates gradually decreased from 0.1819 to 0.0032 in the subsequent years. In the sixth follow-up year, the outpatient expenses of the intervention group were significantly lower than those of the comparison group (β= -0.028; *P* = 0.0078). Similar results were observed for total health care expenses. The reduction in expenses occurred in the fifth follow-up year and became significant in the sixth follow-up year (β= -0.0336; *P* = 0.0103). The DM P4P program contributed to the reduction in total care expenses in the intervention group after 5 years of follow-up.

To measure the spillover effect of the DM P4P program on depressive symptoms in mental health-related care not covered by program indicators, we extended the analysis to the “third” difference in expenses between the occurrence and absence of depression based on the DID model. Table 4 presents the results where triple-difference models were used to analyse the health care expenses of outpatient care and overall health care expenses for diabetes patients with and without new depressive symptoms. For diabetes patients with depressive symptoms, those enrolled in the P4P program had lower outpatient expenses than those not enrolled in the program. Net effects were observed over the follow-up years, and the estimates showed significant differences from the third follow-up year. With regard to total health care expenses, the same trends were observed in model estimates between the intervention and comparison groups for all six follow-up years. People with diabetes who developed new depressive symptoms in the intervention group showed decreased total health care expenses compared with people with diabetes who developed new depressive symptoms in the comparison group. This reduction became significant at the sixth follow-up year (β= -0.0749; *P* = 0.0185).

**Table 4:** Estimations of the impact of the DM P4P program on health care expenses of patients with and without depressive symptoms based on the difference-in-differences-in-differences method

		**Outpatient Expenses**								**Total Expenses**					
Follow-up		Estimate		SE		*P* Value				Estimate		SE		*P* Value	
Year 1		-0.0843		0.0768		0.2725				-0.049		0.0846		0.5622	
Year 2		-0.0704		0.0458		0.1239				-0.0418		0.0541		0.4399	
Year 3		-0.0761		0.0348		0.0287				-0.0546		0.0402		0.1742	
Year 4		-0.0981		0.0305		0.0013				-0.0495		0.0349		0.1564	
Year 5		-0.1058		0.0285		0.0002				-0.0632		0.0331		0.0564	
Year 6		-0.1055		0.0273		0.0001				-0.0749		0.0318		0.0185	

### Depression-related care expenses of diabetes patients with depressive symptoms between the intervention and comparison groups

Table 5 shows the results of further analyses of the health care expenses of diabetes patients with depressive symptoms between the intervention and comparison groups. Outpatient and total expenses for depression-related care were further analysed.

**Table 5:** Expenses of diabetes patients with depressive symptoms between the intervention and comparison groups

	**Depression-related Outpatient Costs**				**Depression-related Total Costs**		
Follow-up	Estimate	SE	P Value		Estimate	SE	P Value
Year 1	-0.0999	0.1607	0.534		-0.4858	0.1869	0.0093
Year 2	-0.3112	0.1264	0.0138		-0.3303	0.1337	0.0135
Year 3	-0.2493	0.1098	0.0231		-0.4511	0.1137	< 0.0001
Year 4	-0.2557	0.0921	0.0055		-0.1756	0.0951	0.0647
Year 5	-0.1951	0.0836	0.0196		-0.2022	0.0856	0.0182
Year 6	-0.1998	0.0784	0.0108		-0.2122	0.0801	0.0081

Diabetes patients with depressive symptoms enrolled in the DM P4P program showed lower depression-related outpatient expenses, with significant differences from the second follow-up year. Similar differences were observed for the total depression-related health care expenses. Diabetes patients with depressive symptoms enrolled in the DM P4P program had lower expenses for depression-related care than those not enrolled in the program.

## Discussion

The main objective of this study was to assess whether the DM P4P program had spillover effects on diabetes patients with psychological complications such as depressive symptoms in Taiwan. The current DM P4P programs focus on the physiological care of diabetes patients, and several studies have shown improved outcomes in diabetes patients [7, 18, 19, 22]. This study analysed the occurrence of depressive symptoms and related health care expenses of diabetes patients with depressive symptoms to evaluate the impact of the DM P4P program on depression symptoms. The results demonstrated that the DM P4P program benefitted diabetes patients with comorbid depressive symptoms, while psychological disease was not covered in the incentive scheme of the program. The rollout of this program not only improved diabetes outcomes but also increased physicians’ attention to psychosocial problems and reduced health care expenses for diabetes patients with depressive symptoms. The spillover effect of the DM P4P program on nondiabetic conditions provided evidence of a modest cost-saving effect of the diabetes care program in the context of depressive symptoms, a common mental disorder symptom in patients with diabetes.

Previous research has shown that the DM P4P program has a positive impact on physiological care outcomes and expenses for people with diabetes. Pay-for-performance programs may reduce the likelihood of diabetes-related hospitalizations and emergency department visits among diabetes patients with and without hypertension [18]. Patients who participated in the DM P4P program had a lower risk of cancer-related mortality, annual mortality and heart failure than those who did not [22]. Another study showed that diabetes patients enrolled in the P4P program had significantly lower overall health care expenses than their counterparts during follow-up [19]. This study examined the long-term effects of the DM P4P program using nationwide longitudinal cohort data. The results indicated that patients in the intervention group had higher outpatient and total expenses in the early follow-up years, but the differences became nonsignificant and then became lower than those of unenrolled patients in the sixth follow-up year. Considering that some of the DM P4P financial incentives are based on certain process indicators, such as required blood tests and health education, the initial higher expenses in the intervention group may be due to increased density of care with regular follow-up visits. The program had a positive impact on controlling disease progression and complications, eventually reducing health care expenses. The findings from this study are similar to those in previous studies [4, 19].

The risk of developing depressive symptoms is higher in diabetes patients than in the general population. Health outcomes in diabetes patients with comorbid depression may be worsened by the interaction of conditions [35–37]. Associated physical and psychological complications lead to increased health care utilization and financial burdens on the health care system [38]. Therefore, the awareness and detection of depressive symptoms by physicians during follow-up visits are important [39]. In this study, we found a significant difference in the incidence of depressive symptoms between the intervention and comparison groups. Taiwanese people have a high level of accessibility to health care services without compulsory referrals. A previous study revealed that 83% of patients perceived good accessibility to care under the Taiwanese health care system [40]. However, only 27% of patients with depression had initial contact with their health providers, and this situation may be due to stigma or lack of awareness [40]. This study showed that diabetes patients enrolled in the DM P4P program had a higher percentage of depressive symptoms from the second year of follow-up. Since patients in both groups had no medical claims for depressive symptoms before enrolling in the program, the difference in the occurrence of depressive symptoms may be attributable to more regular follow-up visits and increased physician attention facilitated by the DM P4P program.

Unintended consequences of P4P programs, such as spillover effects, may become an important aspect of disease management and program evaluation. This is especially true given the rising health care expenses of chronic disease patients with complications, and positive spillover effects were observed on nonincentivized factors in the intervention groups [41]. In the UK, incentives were designed for health care providers to improve certain targeted indicators. Researchers found that providers would make investments in quality out of the P4P scheme to achieve stricter incentivized factors with greater rewards [41]. Moreover, a possible spillover effect on medical cost savings due to diabetes-related hospitalizations and non-diabetes-related outpatient visits was identified in the Taiwanese diabetes P4P program [19]. In this study, we analysed the long-term effects of the DM P4P program on the health care expenses of diabetes patients with new depressive symptoms in the intervention and comparison groups. For diabetes patients with new depressive symptoms, those who continuously remained in the P4P program had significantly lower outpatient and total health care expenses than patients who were not enrolled in the program. Moreover, diabetes patients with new depressive symptoms who participated in the program had lower health care expenses for depression-related care than unenrolled patients with new depressive symptoms. Regular follow-up visits and better health outcomes for patients enrolled in the DM P4P program may lead to higher patient trust, which may facilitate physicians’ attention to patients with depressive symptoms. The current incentive indicators of the DM P4P program focus on the physiological care measures of diabetes. Most previous studies have shown the impact of the P4P program only on diabetes-related physiological care. In this study, the DM P4P program extended the benefits to mental health care, such as depression management, and lowered accompanying health care expenses. The results indicated that the DM P4P program had positive spillover effects on nonincentivized items such as depression management.

### Limitations

This study has some limitations. First, the enrolment of patients into the P4P program was decided by the participating physicians, and selection bias may exist. This study employed PSM to reduce the selection effect and improve the covariate distribution balance, increasing comparability between the patients in the intervention and comparison groups. However, certain unobserved factors cannot be controlled for, such as health literacy or the willingness of patients to engage. DID methods were implemented to control for unobserved factors that could bias the estimate of the causal effect. Second, the outcome variable for depressive symptoms was defined by outpatient visit diagnoses or antidepressant use in the NHI claims data. Incomplete diagnostic assessments and therapeutic undertreatment were found in outpatient settings [30]. The incidence of depression may be underestimated because of nonpsychiatric diagnoses or the social stigma surrounding depression. The DID and triple-difference models can control for unobserved variables and produce unbiased estimations of program effects with the incorporation of covariates [42]. The key assumption in DID models is the parallel trend assumption that the outcomes of the intervention and comparison groups would have evolved similarly in the absence of the program intervention. We consider that the degree of underdiagnosis might be similar in the intervention and comparison groups at baseline, which may lessen the influence on our study results. Since there is no statistical test for this assumption, the limited observation length of the preintervention period may have an impact on the validation of the parallel trends test [43]. A study indicated that the DID model with PSM methods showed outstanding performance in estimating program effects when the parallel trends assumption was violated [44]. Finally, the Taiwanese health care system is characterized by high accessibility without long waiting times to see health care providers contracted with the NHI and has no compulsory referral requirement. This may limit the generalizability of these findings to other health care systems.

## Conclusions

Rising health care costs related to chronic diseases are a global challenge. Previous studies have shown positive intended effects of the DM P4P program in Taiwan, such as better adherence to practice guidelines, better health outcomes and lower health care expenses, but these studies focused on physiological outcomes associated with diabetes. This study revealed that the DM P4P program has positive spillover effects on nonincentivized psychological care. The DM P4P program may benefit people with diabetes by screening for depressive symptoms and lowering accompanying health care expenses. The positive spillover effects may be an important aspect of physical and mental health in patients with chronic diseases enrolled in disease management programs while contributing to the control of health care expenses for chronic diseases. The DM P4P program with incentive indicators designed mainly for the physiological care of diabetes can have a good control effect on the medical expenses of diabetes and its psychological complications at the same time and may even further improve the quality of medical care. This finding has value in driving expanded enrolment of people with diabetes in the DM P4P program and revising future incentive indicators in P4P programs. Future studies may examine the causal and cost-saving effects with a longer follow-up to provide more solid evidence for spillover effects on nonincentivized health care services.

## Data Availability

The data used in this study were retrieved from the medical claims database of Taiwan’s National Health Insurance Administration. All data generated or analysed during this study are included in this published article. The database cannot be made available upon reasonable request or in a public repository due to the Personal Data Protection Act.

## Abbreviations

DM: Diabetes mellitus
P4P: Pay-for-performance
NHI: Taiwan National Health Insurance
ICD-9-CM: International Classification of Diseases, Ninth Revision, Clinical Modification
PSM: Propensity score matching
CCI: Charlson Comorbidity Index
DCSI: Diabetes Complications Severity Index
DID: Difference-in-differences
Triple-difference: Difference-in-difference-in-differences
SMD: Standardized mean difference
